# Adaptation of Rice to the Nordic Climate Yields Potential for Rice Cultivation at Most Northerly Site and the Organic Production of Low-Arsenic and High-Protein Rice

**DOI:** 10.3389/fpls.2020.00329

**Published:** 2020-04-30

**Authors:** Mingliang Fei, Yunkai Jin, Lu Jin, Jun Su, Ying Ruan, Feng Wang, Chunlin Liu, Chuanxin Sun

**Affiliations:** ^1^Key Laboratory of Crop Epigenetic Regulation and Development in Hunan Province, Hunan Agricultural University, Changsha, China; ^2^Department of Plant Biology, Uppsala BioCenter, Linnean Centre for Plant Biology, Swedish University of Agricultural Sciences, Uppsala, Sweden; ^3^Key Laboratory of Education, Department of Hunan Province on Plant Genetics and Molecular Biology, College of Bioscience and Biotechnology, Hunan Agricultural University, Changsha, China; ^4^Institute of Biotechnology, Fujian Academy of Agricultural Sciences, Fuzhou, China; ^5^College of Agronomy, Hunan Agricultural University, Changsha, China

**Keywords:** low-arsenic rice, high-protein rice, infant food, rice for cold climate, Uppsala rice cultivation, Baltic Sea

## Abstract

There is an urgent demand for low-arsenic rice in the global market, particularly for consumption by small children. Soils in Uppsala, Sweden, contain low concentrations of arsenic (As). We hypothesize that if certain *japonica* paddy rice varieties can adapt to the cold climate and long day length in Uppsala and produce normal grains, such a variety could be used for organic production of low-arsenic rice for safe rice consumption. A *japonica* paddy rice variety, “Heijing 5,” can be cultivated in Uppsala, Sweden, after several years’ adaptation, provided that the rice plants are kept under a simple plastic cover when the temperature is below 10°C. Uppsala-adapted “Heijing 5” has a low concentration of 0.1 mg per kg and high protein content of 12.6% per dry weight in brown rice grain, meaning that it thus complies with all dietary requirements determined by the EU and other countries for small children. The high protein content is particularly good for small children in terms of nutrition. Theoretically, Uppsala-adapted “Heijing 5” can produce a yield of around 5100 kg per ha, and it has a potential for organic production. In addition, we speculate that cultivation of paddy rice can remove nitrogen and phosphorus from Swedish river water and reduce nutrient loads to the Baltic Sea and associated algae blooms.

## Introduction

Paddy rice is the staple food for a large proportion of the human population, especially for Asian people. The global production of rice in 2017 was nearly one billion tons, with the top three producers being China, India, and Indonesia (Food and Agricultural Organization of the United Nations (FAO), 2017). Some southern European countries, such as Italy, Spain, Greece, Portugal, France, and Romania, also produce rice, with total annual production of around 3 million tons, as measured in 2017 (Food and Agricultural Organization of the United Nations (FAO), 2017). Based on the current human population, a rough calculation indicates that the global consumption of rice grains is around 140 kg per capita.

Rice has become the major staple food for humans for perhaps three main reasons: (1) rice grains have good cooking quality, excellent taste, and are easy to digest (Tian et al., 2009; Saikrishna et al., 2018; Ziegler et al., 2018; Bao, 2019; Mestres et al., 2019; Zhou et al., 2020); (2) very few people exhibit allergic reactions to rice grains, and rice is thus often regarded as hypoallergenic (Zhu et al., 2015); and (3) rice has high productivity per unit area, and, in some rice-cultivating zones, up to three/four cultures of rice can be produced annually (Khush, 2003). Thus, paddy rice has come to meet the human food demand and to dominate as a staple food. However, there are several drawbacks associated with paddy rice: (i) cultivation of paddy rice is laborious (Food and Agricultural Organization of the United Nations (FAO), 2019; International Rice Research Institute, 2019); (ii) rice paddies require a good water supply (Sahebi et al., 2018; Mukamuhirwa et al., 2019); (iii) paddy rice agriculture produces the greenhouse gas methane (Su et al., 2015); and (iv) rice grains can contain high concentrations of metals if the cultivation environment or soil are polluted (Rahman et al., 2014; Singh et al., 2015; Awasthi et al., 2017; Chen et al., 2017; Norton, 2019).

There has been an intensive search for solutions to these problems in recent years. As mechanization and automation in crop production increase, cultivation of paddy rice has become easier (Food and Agricultural Organization of the United Nations (FAO), 2019; International Rice Research Institute, 2019). Plant researchers are actively working to develop paddy rice with drought tolerance (Sahebi et al., 2018; Mukamuhirwa et al., 2019) and low methane emission (Su et al., 2015). Attempts to lower the metal concentration in rice grains are also under way. High concentrations of metals, such as arsenic (As), mercury (Hg), and cadmium (Cd), in rice grains can result in chronic poisoning and can lead to impaired human health (Fontcuberta et al., 2011; Rahman et al., 2014; Huang et al., 2015; Singh et al., 2015; Awasthi et al., 2017; Chen et al., 2017; Li et al., 2018; Norton, 2019). The actual concentrations of metals in rice grain depend on the amount of metals in the cultivation environment and in the soil (Bogdan and Schenk, 2009; Satpathy et al., 2014; Zhao et al., 2015; Rahman et al., 2018) as well as differences in uptake between rice genotypic variations (Norton et al., 2014; Yan et al., 2019). Organic cultivation in relatively natural conditions on less contaminated soil can result in lower metal concentrations in grain (Satpathy et al., 2014; Zhao et al., 2015). However, recent reports in Sweden indicate that, surprisingly, organic rice on the Swedish market contains significant amounts of As (Radio Sweden, 2015a; The Local Europe AB, 2015). This high As concentration poses a particular threat to the health of small children in kindergartens in Sweden, as organic rice cakes are often served as a snack in these establishments (Radio Sweden, 2015b). On the European and global markets, low-arsenic rice is also in high demand for consumption (Kollander and Sundström, 2015; U.S. Food and Drug Administration, 2016) and particularly for infant food (Juskelis et al., 2013; Houlihan, 2017).

## Materials and Methods

### Rice Materials and Cultivation Conditions

Over 20 rice lines, varieties, or cultivars were collected from Northeastern China to be screened for cultivating adaptations (Supplementary Table S1). “Nipponbare” was used as a control line. Rice seeds were germinated on water-submerged filter papers at 28°C in darkness for 4 days (Supplementary Figure S1A). Germinated seeds were transferred individually to a pot (10 × 10 cm on top and 7 × 7 cm on bottom with a height of 7.5 cm) with organic soil (called “såjord/seed compost” containing at least perlite, cow manure, lime, and natural humus; Hasselfors Garden, Örebro, Sweden) and cultivated in a greenhouse (Supplementary Figure S1B). Cultivating conditions of the greenhouse were 16 h light and 8 h dark at 30°C/15°C with a constant humidity of 80% and light intensity of 300 μmol per m^–2^ s^–1^. After 10 days’ cultivation in the greenhouse, seedlings at a stage of *ca* three leaves were transplanted to open fields. For screening of adaption, the cultivation of different lines was not covered. For a production test, “Heijing 5” was covered with a simple plastic polytunnel in the fields when the temperature was below 10°C (Supplementary Figure S1). Field cultivations were performed for ca 4.5 months with normal farming practices before harvesting. Paddy irrigation was mainly dependent on rainfall, and, when necessary, groundwater was used for the water supply. During cultivation, air and paddy soil temperatures were recorded. “Heijing 5” plants were also cultivated in a greenhouse and a field without a cover as controls. Plants were harvested and stored dry at room temperature for different analyses.

### Determination of Concentrations of Total As, Hg, and Cd

Analysis of total As, Hg, and Cd in raw rice grains, aboveground tissues, and paddy soil/water mixture was done by a commercial service at the company Eurofins Sweden^1^ in an Accredited Laboratory (No. ISO/IEC 17025: 2005 SWEDAC 1125). Briefly, for analysis of total arsenic and cadmium, samples were digested according to the Swedish standard of SS-EN-ISO-13805:2014. Pretreatment was an acid-based microwave digestion at 220°C. A digested sample was first diluted to a total volume of 50 ml, and then additional dilution was completed on the instrument prior to analysis. Quality assessment of the digestion is based on a blank sample and CRM (certified reference materials) that are analyzed in every batch. Dilution was controlled by an internal standard, and the dilution was added to each sample during the pretreatment. Determination of total As and Cd was carried out by Inductively Coupled Plasma Mass Spectrometry (ICP-MS) according to SS-EN-ISO 17294: 2016. Calibration was performed using calibration standards. Quality assurance consisted of blank samples, synthetically prepared QC samples (ICV – initial calibration verification), as well as CRM (certified reference materials), which were analyzed periodically throughout the sample batch to verify instrument’s stability. An internal standard was added inline during the analysis to monitor the conditions in the plasma and account for possible matrix effects. For mercury analysis, digestion of samples was according to the Swedish SS-EN16277:2012 annex D. Samples were pretreated for analysis by addition of HNO, HCl, and H_2_O_2_ and digested in open vessels under heating. The temperature program ranged from 60 to 120°C for a total of 2.5 h. One blank and one control sample (verified reference materials) were included in every batch. Analysis of total mercury was performed by AFS (PS Analytical). Synthetic solutions are included as standards and QCs in every instrument run.

### Determination of Protein Content in Rice Grains

Rice grains were grounded and dried completely. The protein content was determined in an accredited Laboratory at the Swedish University of Agricultural Sciences (No. ISO 14001) according to the Kjeldahl method following a protocol described in Nordic Committee on Food Analysis (Nordic committee on food analysis, 1976).

### Determination of Starch and Amylose Content in Rice Grains

Rice grains were grounded and dried completely. The starch and amylose content were analyzed by Megazyme kits for total starch and amylose/amylopectin, respectively (Megazyme, Bray, Co. Wicklow, Ireland), and as described in Jin et al. (2017a) and Sun et al. (2005).

### Thin Layer Chromatography Analysis and Oil Content Determination

Thin Layer Chromatography analysis and oil content determination on Gas Chromatography (GC) were performed according to Aslan et al. (2015) and Jin et al. (2017b).

### Agronomic Trait Investigations and Weather Conditions

After maturity, 48 rice plants were randomly selected from two paddy plots for agronomic trait investigations. Weather conditions in Uppsala, Sweden, and Heihe, China, for 2018 were obtained from different meteorology websites.

### Statistical Analysis

Biological and technical triplicates were used in all experiments except analysis of starch and amylose in which technical duplicates were used according to the manufacturer’s protocols (Megazyme, Bray, Co. Wicklow, Ireland). One-way ANOVA was used for analysis as described previously (Jin et al., 2017a).

## Results and Discussion

### Cultivation of Paddy Rice at High Latitudes

Soils in Uppsala, Sweden, contain a low concentration of As (Ljung et al., 2006). The concentration ranges between 1 and 6 mg kg^–1^ with a median concentration of *ca* 3.5 mg kg^–1,^ and this is mainly due to geological sources (Ljung et al., 2006). The concentration is low compared with the levels in other geological countries that are normally over 8 mg kg^–1^ (Bogdan and Schenk, 2009; Camacho et al., 2011; Kong et al., 2018; Rahman et al., 2018). Since As concentration in paddy soils significantly impact As concentrations in rice grains (Bogdan and Schenk, 2009; Rahman et al., 2018), we hypothesize that production of rice in Uppsala fields would be a potential alternative to produce low As rice that meets the statutory requirements for rice consumed in Sweden and other countries. The challenge, however, is to identify a rice variety that could adapt to the summer climate in central Sweden (i.e., mild temperature and long days) and produce full grains. If such a variety could be identified, it could be used in a test of organic production of rice for a low As concentrations.

**FIGURE 1:**
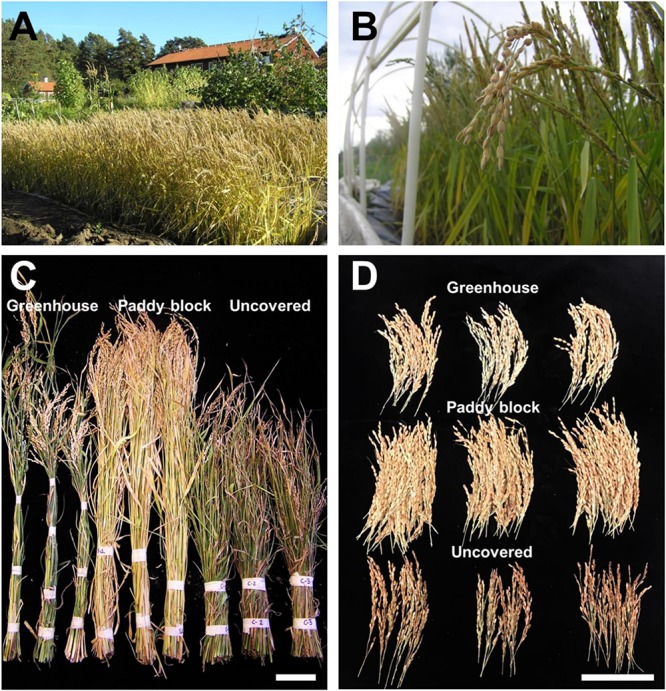
A Chinese paddy rice variety “Heijing 5” (*japonica*) adapted to the Uppsala climate and produced normal panicles with fully-filled grains. **(A)** “Heijing 5” paddy in Uppsala. **(B)** “Heijing 5” panicles in a paddy in Uppsala. **(C)** Harvested rice plants from: (left) a greenhouse in Uppsala, (center) a paddy covered with a simple and easily openable polytunnel when the temperature was below 10°C, and (right) a paddy without a cover. **(D)** Harvested panicles from: (top row) a greenhouse in Uppsala (middle row), a paddy covered with plastic when the temperature was below 10°C, and (bottom row) a paddy without a cover. Bars = 10 cm in **(C,D)**.

**FIGURE 2:**
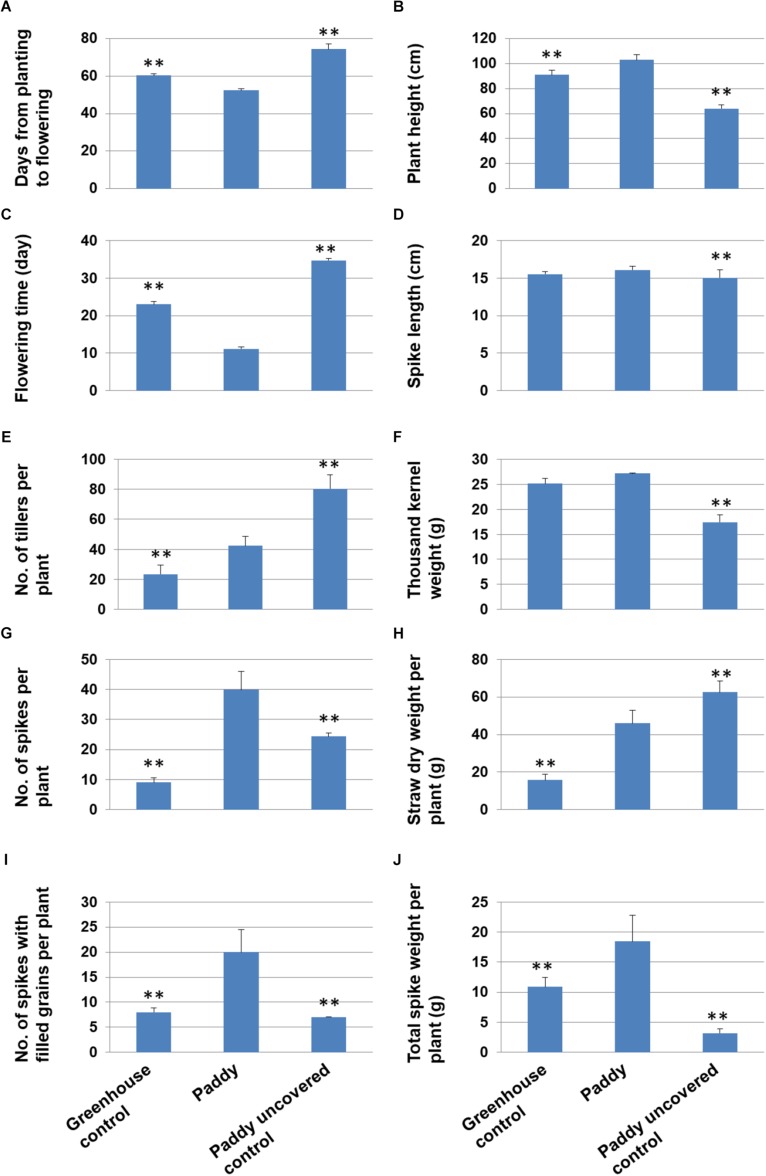
**(A–J)** Phenotypical traits of “Heijing 5” plants cultivated in Uppsala in (left) a greenhouse control, (center) a paddy block covered with a polytunnel shed when air temperature was below 10°C, and (right) an uncovered paddy block control. One-way ANOVA was used (Error bars show s.d.). ^∗∗^*P* < 0.01 is shown for significant differences between controls and paddy.

Paddy rice may be able to adapt to cold climates (Falk and Andrus, 2012; Cruz et al., 2013). It could thus potentially be cultivated in countries with a cold climate, such as in Sweden. However, it is unknown whether paddy rice can adapt to the long day length in Uppsala, Sweden (59.86° N, 17.64° E). In 2012, we initiated a trial on whether certain paddy rice varieties could adapt to the Uppsala climate and produce full grains. We collected 24 varieties of *japonica*-type paddy rice from Northeastern China (Supplementary Table S1), which has a relatively similar climate to central Sweden, and cultivated them in a field in Uppsala. During the trial, we observed that half the varieties were able to flower but without normal grain filling in all except one variety, “Heijing 5,” which produced partly filled grains. Using the partly filled grains, we cultivated “Heijing 5” in a field in Uppsala for 5 years consecutively (2013–2017), and we observed that grain filling over the generations improved year on year. In 2018, we used grains from the Uppsala-adapted “Heijing 5” in a field trial in Uppsala to assess metal uptake and other quality traits. Since rice productivity is dependent on an accumulated temperature (Wang et al., 2015), we used a simple and easily openable polytunnel to cover the growing rice plants when the air temperature was forecast to fall below 10°C (Supplementary Figure S1). Using a potocol, we developed for rice cultivation in Uppsala (Supplementary Figure S1), we successfully produced normal rice plants with fully-filled grains (Figure 1). When we examined important agronomic traits, we observed that the productivity-dependent traits, i.e., spike length, thousand kernel weight, number of spikes with filled grains, and total spike weight per plant, were significantly better than in an uncovered control and similar or slightly better than in a greenhouse control (Figure 2 and Supplementary Dataset S1). Based on spike productivity of 18.5 g per plant (Figure 2J), the Uppsala-adapted “Heijing 5” would theoretically produce around 5100 kg per ha, which is a normal yield for a conventional paddy rice variety (Food and Agricultural Organization of the United Nations (FAO), 2017). It is obvious that “Heijing 5” in Uppsala has a short life period (Figures 2A,C) with a quite normal trait related to yield (Figures 2D,F,I,J). We do not understand the mechanism as to how a normal yield trait was generated within a short growth period, but the high temperature difference between day and night in Uppsala may help biomass accumulation. An in-depth study on the mechanism in molecular biology is needed for the further breeding and improvement of the rice in Uppsala.

### Concentrations of Metals in Rice Grain

**FIGURE 3:**
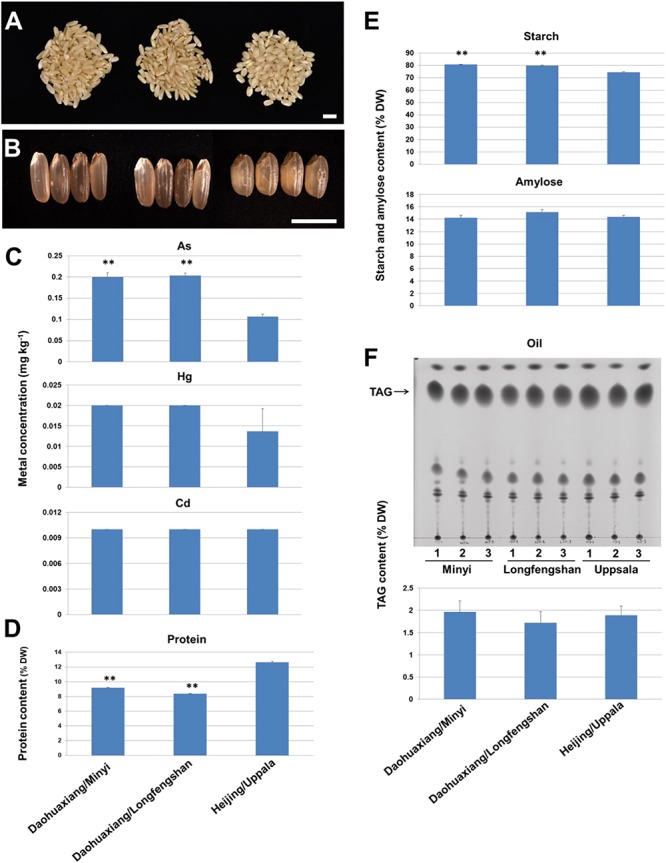
Concentration or content of metals, proteins, starch, amylose, and oil in paddy rice cultivars grown in China and Sweden. **(A,B)** Raw rice caryopses of a purchased Chinese cultivar “Daohuaxiang” (*japonica*) cultivated in (left) Minyi and (center) Longfengshan in Wuchang City, China, and (right) “Heijing 5” cultivated in Uppsala. **(C)** Concentration of arsenic (As), mercury (Hg), and cadmium (Cd), and **(D)** Content of proteins in the corresponding raw rice grains. **(E)** Content of starch and amylose. **(F)** Content of oil or triacylglycerol (TAG). Upper panel in **(F)** is a Thin Layer Chromatogram (TLC). Bars = 6 mm in **(A,B)**. One-way ANOVA was used (Error bars show s.d.). ^∗∗^*P* < 0.01 is shown for significant differences between “Daohuaxiang” and “Heijing 5.”

On examining metal concentration and other quality traits in Uppsala-adapted “Heijing 5,” we observed that the As concentration was significantly lower than in a well-known organic rice cultivar (“Daohuaxiang”) from the Chinese market, which was produced in Minyi and Longfengshan, Wuchang city (Figures 3A–C). The As concentration in Uppsala-adapted “Heijing 5” raw grains or brown rice was around 0.10 mg per kg (Figure 3C and Supplementary Dataset S1), which is below the threshold set for all rice food applications by European Union institutions and bodies (e.g., 0.25 mg per kg for raw rice grains and 0.10 mg per kg for infant food) (Access to European Union law, 2006). Interestingly, Uppsala-adapted “Heijing 5” had a higher protein content and lower starch content than “Daohuaxiang” (Figures 3D,E and Supplementary Dataset S1), resulting in higher nutritional value, particularly for small children. The oil content was around the same as in “Daohuaxiang” (Figure 3F and Supplementary Dataset S1). Its eating quality will be tested in Sushi food.

The concentration of As in Uppsala soils is low, and Hg and Cd are within the agricultural ranges or a little above with median concentration of 3.46–3.91 mg kg^–1^ for As, 0.212–0.218 mg kg^–1^ for Cd, and 0.130–0.146 mg kg^–1^ for Hg in 0–20 cm-deep soil (Klang and Eriksson, 1997; Tsadilas et al., 2005; Ljung et al., 2006; Gan et al., 2018). We did not observe a difference in Hg and Cd concentration between Uppsala “Heijing 5” and “Daohuaxiang” from Minyi and Longfengshan, and this was probably due to the normal concentration of Hg and Cd in Uppsala Soils. The floodwater was mainly based on rainfall and ground water supply. Since concentrations of As, Hg, and Cd in the ground water in Uppsala close to Lake Mälaren and Stockholm are very low, with median values of *ca* 0.54 μg L^–1^ for As, 15.6 ng L^–1^ for Hg, and 0.05 μg L^–1^ for Cd (Aastrup and Thunholm, 2001), and the water is even drinkable after softening (Uppsala Vatten and Avfall AB, 2020), we suggest that the irrigation may have little effect on final concentrations of the metals in the rice grains.

**FIGURE 4:**
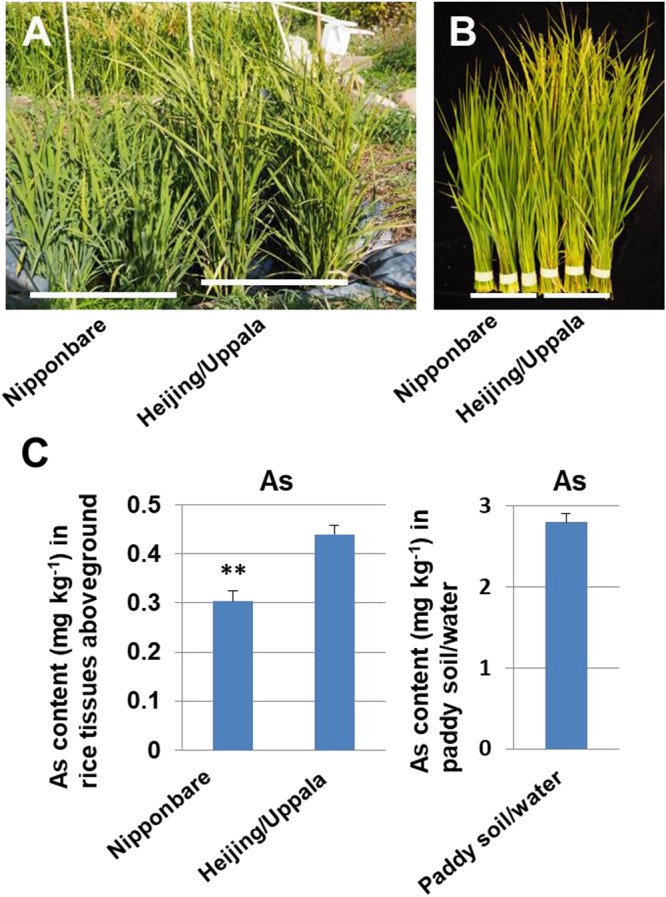
Concentration of As in rice tissues aboveground and paddy soil/water mixture. **(A)** Rice plants of “Nipponbare” and “Heijing 5” in an Uppsala paddy plot. **(B)** Harvested rice tissues aboveground of “Nipponbare” and “Heijing 5” from a paddy plot. **(C)** As concentration in rice tissues aboveground of “Nipponbare” and “Heijing 5” and As concentration in Uppsala paddy soil/water mixture. One-way ANOVA was used (Error bars show s.d.). ^∗∗^*P* < 0.01 is shown for significant differences between “Nipponbare” and “Heijing 5.”

To support the concept that the low As concentration in Uppsala “Heijing 5” grains was caused by the low concentration of Uppsala soils, not by genotypic variations, we cultivated “Nipponbare” and “Heijing 5” in the same plot and measured As concentration in aboveground tissues of both genotypic rice (Figure 4). The result showed that the As concentration in “Nipponbare” aboveground tissues was significantly lower than “Heijing 5” (Figure 4C and Supplementary Dataset S1), meaning that “Nipponbare” uptakes less As from soil/water than “Heijing 5.” However, the As concentration of 0.30 mg kg^–1^ in “Nipponabre” straws and 0.44 mg kg^–1^ in “Heijing 5” in our experiment (Figure 4C) is very low compared with a normal/typical straw As concentration for “Nipponbare” having *ca* 5 mg kg^–1^ (Ma et al., 2008; Ye et al., 2017). We have suggested that the low As concentration in the aboveground tissues of both “Nipponbare” and “Heijing 5” may result from a low concentration in the paddy soil/water. To support the suggestion, we determined the total As concentration in the plot soil/water. The result showed that an average of 2.8 mg kg^–1^ in triplicate samples was obtained (Figure 4C). The concentration is low compared with normal paddy soil concentration above 8.3 mg kg^–1^ (Bogdan and Schenk, 2009; Rahman et al., 2018). Norton (2019) suggested a number of transporters are involved in As uptake and translocation in rice. However, how those transporters respond to temperatures is basically unknown. A study on how a low temperature affects the transport efficiency of those transporters is clearly needed in future.

### Temperature Requirement for Rice Cultivation at High Latitudes

We successfully demonstrated that a *japonica* rice variety, “Heijing 5,” can be adapted to the Uppsala climate and produce a normal yield of grain. “Heijing 5” was originally developed in Heihe, China (50.52° N, 127.53° E), a region with a similar climate to Uppsala, Sweden (Supplementary Figure S2). To our knowledge, Uppsala is the most northerly site at which grain-filling rice has been produced to date. It was reported that rice was successfully cultivated in the Fraser valley in Abbotsford, Canada (Philippsen, 2015). However, Abbotsford has latitude of *ca* 49.03° N, which is similar to Heihe, China (Supplementary Figure S2). “Heijing 5” needs an accumulated temperature of 2100 degrees above 10°C for normal productivity (Wang, personal communication). On comparing the climate in Uppsala and Heihe, we noted that the climate in Uppsala is slightly colder than in Heihe, with the average temperature in Uppsala reaching around 10°C in May (Supplementary Figure S3 and Supplementary Dataset S1), which covers a corresponding stage of rice early seedlings (Supplementary Figure S1C). Thus, at higher latitudes it may be necessary to cover rice seedlings in May if the temperature is below 10°C, to ensure that “Heijing 5” can achieve sufficient accumulated temperatures for normal productivity. A larger production test in 2019 indicated that the same trait of fully filled grains can be achieved when the cultivation conditions were applied to “Heijing 5” cultivation in Uppsala.

### Environmental Benefits

Paddy rice utilizes nitrogen and phosphorus in water as a source of nutrients, and cultivation of paddy rice can thus remove excess nitrogen and phosphorus from water. In recent years, the nitrogen and phosphorus concentration in many Swedish rivers has been increasing, contributing to algae blooms in the Baltic Sea (Ahtiainen et al., 2014). A preliminary pot experiment indicated that “Heijing 5” can grow normally, without any added fertilizers, in water from the river Fyrisån in Uppsala. This indicates that cultivation of paddy rice in water from Swedish rivers could ultimately reduce the nitrogen and phosphorus load to the Baltic Sea. One positive is that metal concentrations in the river Fyrisån are low (Uppsala Vatten and Avfall AB, 2020): for example, *ca* 0.18 μg L^–1^ for As (Huser et al., 2011). Thus, cultivation of rice using the river water would not generate a high metal concentration in rice grains. In addition, in a breeding program of low-methane rice, “Heijing 5” interestingly displayed a low-methane trait. Cultivation of “Heijing 5” in Uppsala would not release much of the greenhouse gas methane.

Overall, paddy rice cultivation in Uppsala would bring dual benefits: (i) providing rice grains with less As and more protein for food uses, particularly infant food, and (ii) reducing the pollution load on aquatic environments. In the future, one of our breeding goals is to investigate the molecular mechanism of cold acclimation in “Heijing 5” and further increase its low-temperature tolerance in order to avoid the polytunnel protection when temperature is below 10°C. Another breeding goal is to add perennial trait to “Heijing 5” by crossbreeding to make the variety more suitable to cultivation in the Nordic countries with less laborious field management.

## Data Availability Statement

All datasets generated for this study are included in the article/Supplementary Material.

## Author Contributions

CS designed the study and wrote the manuscript. MF, YJ, LJ, and CS performed the research and analyzed the data. YR, JS, FW, and CL discussed the research and revised the manuscript.

## Conflict of Interest

The authors declare that the research was conducted in the absence of any commercial or financial relationships that could be construed as a potential conflict of interest.

## Footnotes

1 www.eurofins.se

## Supplementary Material

FIGURE S1 Protocol illustrating cultivation of “Heijing 5” in Uppsala. **(A)** Germination of seeds in a plate. **(B)** Development of early small seedlings in greenhouse. **(C)** Early seedlings in the field, covered by a simple and easily openable polytunnel when the temperature was below 10°C. **(D)** Late seedlings at tillering stage. **(E)** Rice plants at flowering stage. **(F)** Ripening rice plants.

FIGURE S2 Map indicating the site in China (Heihe; 50.52°N, 127.53°E) at which “Heijing 5” was developed and the site in Sweden (Uppsala; 59.86°N, 17.64°E) at which “Heijing 5” was adapted to the climate after 5 years of cultivation.

FIGURE S3 Weather conditions in Uppsala, Sweden, and Heihe, China. **(A)** Rainfall in 2018 and historical average. **(B)** Average temperature in 2018 and historical average. **(C)** Day length. Data on rainfall and temperature in Uppsala and Heihe taken from https://www.accuweather.com. Data on Uppsala day length taken from https://www.timeanddate.com/sun/and data on Heihe day length taken from https://richurimo.51240.com.

TABLE S1 Rice lines/Varieties/Cultivars used for screening of adaptation.
