# Supplementary material for: Adaptation of Rice to the Nordic Climate Yields Potential for Rice Cultivation at Most Northerly Site and the Organic Production of Low-Arsenic and High-Protein Rice
Source: Front Plant Sci. 2020 Apr 30;11:329. doi: 10.3389/fpls.2020.00329 (PMC7212348; doi:10.3389/fpls.2020.00329)
Supplement: TABLE S1 — Rice lines/Varieties/Cultivars used for screening of adaptation. [file Data_Sheet_2.pdf]

Adaptation of rice to the Nordic climate yields potential for rice cultivation at most northerly site and organic production of low-arsenic and high-protein rice

Mingliang Fei[1,2,3], Yunkai Jin[2], Lu Jin[1,2,3], Jun Su[4], Ying Ruan[1,3], Feng Wang[4], Chunlin Liu[3,5], and Chuanxin Sun[2*]*

[*]**Corresponding authors:**

Chuanxin Sun

Department of Plant Biology, Uppsala BioCenter, Linnean Centre for Plant Biology, Swedish University of Agricultural Science (SLU), P.O. Box 7080, SE-75007 Uppsala, Sweden

Email: Chuanxin.Sun@slu.se; Phone: +46-18-673252

ORCID

Chuanxin Sun, https://orcid.org/0000-0003-2755-0443

Ying Ruan

Key Laboratory of Crop Epigenetic Regulation and Development in Hunan Province, Hunan Agricultural University, Changsha, 410128, China; Key Laboratory of Education Department of Hunan Province on Plant Genetics and Molecular Biology, College of Bioscience and Biotechnology, Hunan Agricultural University, Changsha 410128, China

Email: yingruan@hotmail.com; Phone: +86-13808480429

[1]Key Laboratory of Crop Epigenetic Regulation and Development in Hunan Province, Hunan Agricultural University, Changsha, 410128, China

[2]Department of Plant Biology, Uppsala BioCenter, Linnean Centre for Plant Biology, Swedish University of Agricultural Sciences, P.O. Box 7080, SE-750 07 Uppsala, Sweden

[3]Key Laboratory of Education Department of Hunan Province on Plant Genetics and Molecular Biology, College of Bioscience and Biotechnology, Hunan Agricultural University, Changsha 410128, China

[4]Institute of Biotechnology, Fujian Academy of Agricultural Sciences, Fuzhou 350003, China

[5]College of Agronomy, Hunan Agricultural University, Changsha, 410128, China

Fei et al., 2020. Supplementary Figures S1-S3 and Supplementary Table S1

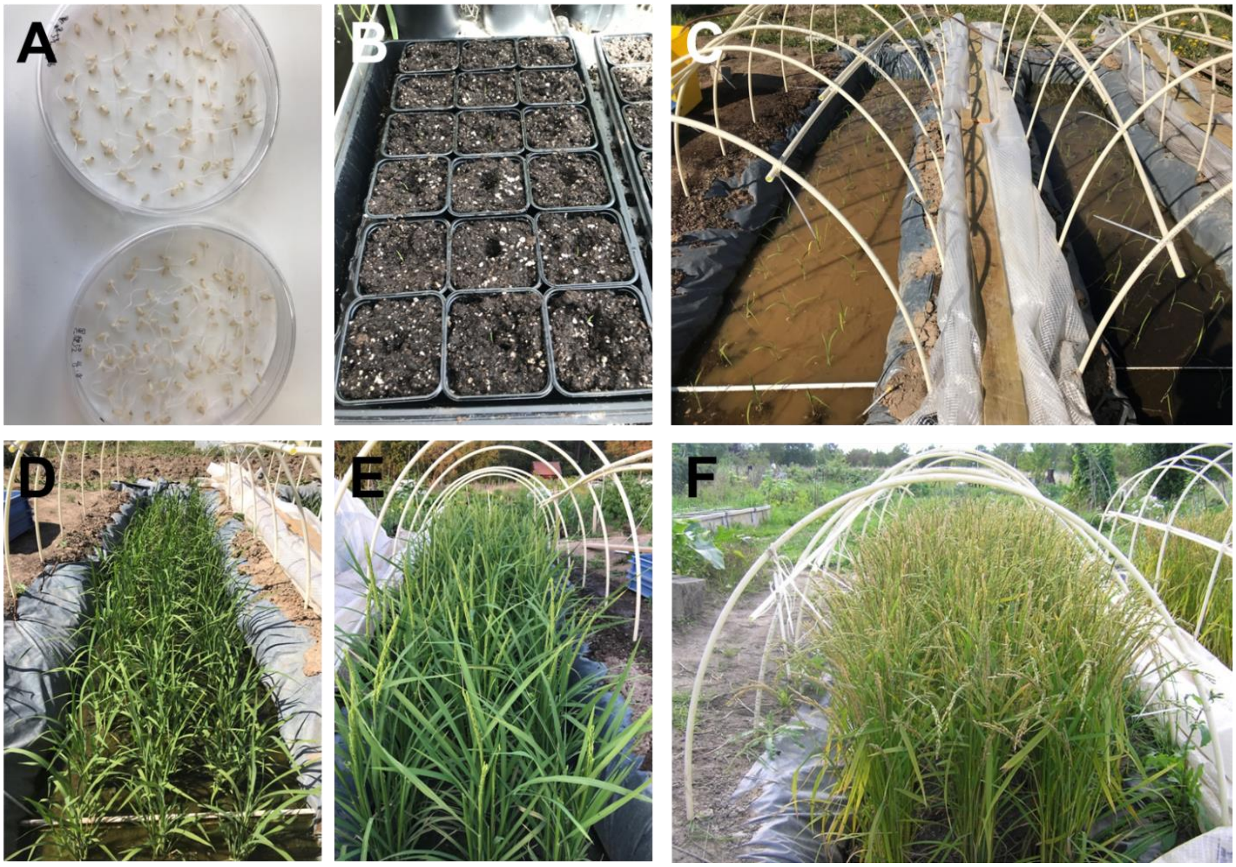

Figure S1 | Protocol illustrating cultivation of 'Heijing 5' in Uppsala. **(A)** Germination of seeds in a plate. **(B)** Development of early small seedlings in greenhouse. **(C)** Early seedlings in the field, covered by a simple and easily-openable polytunnel when the temperature was below 10 °C. **(D)** Late seedlings at tillering stage. **(E)** Rice plants at flowering stage. **(F)** Ripening rice plants.

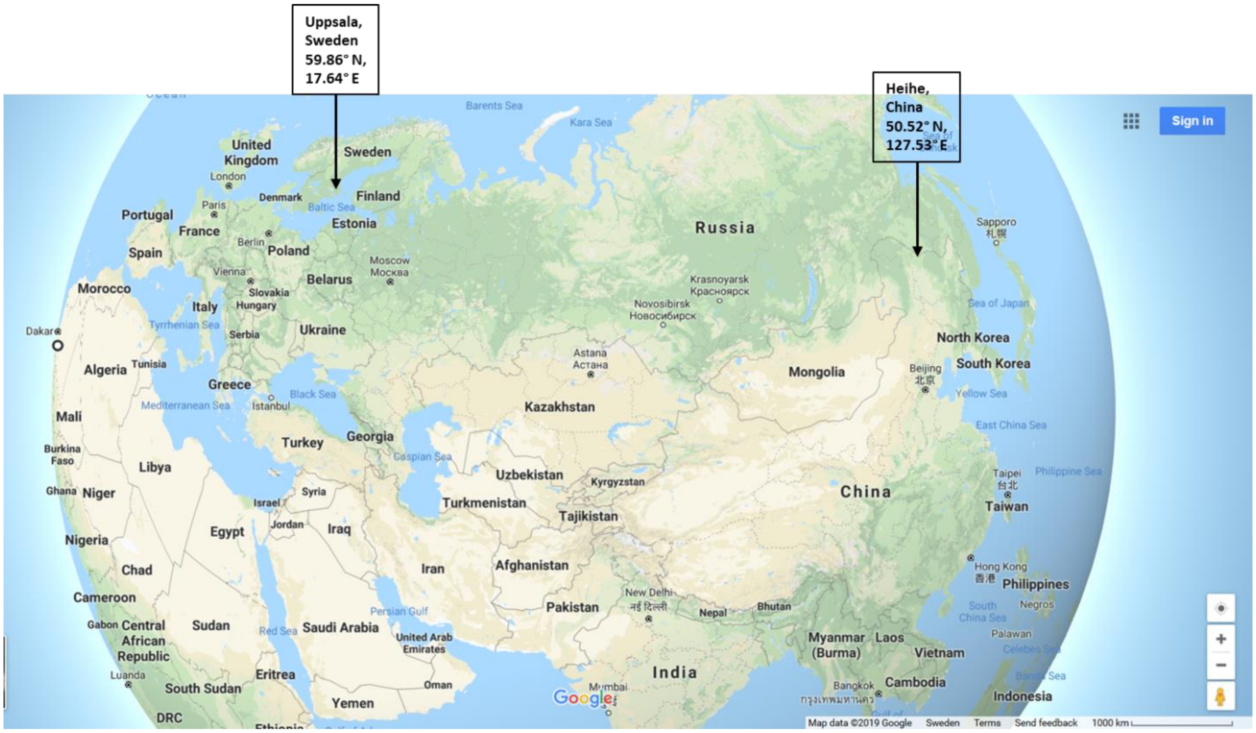

Figure S2 | Map indicating the site in China (Heihe; 50.52°N, 127.53°E) at which 'Heijing 5' was developed and the site in Sweden (Uppsala; 59.86°N, 17.64°E) at which 'Heijing 5' was adapted to the climate after five years of cultivation.

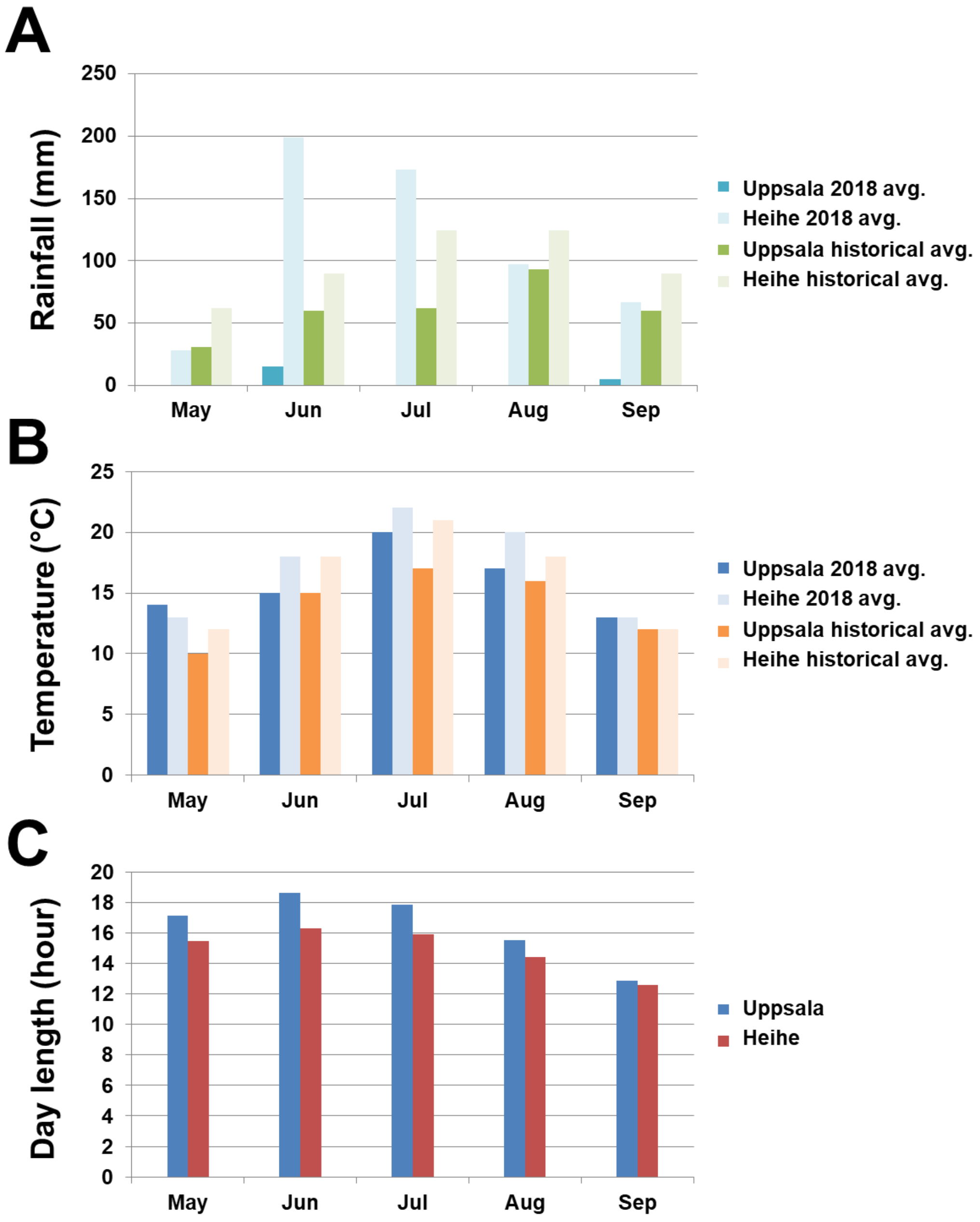

Figure S3 | Weather conditions in Uppsala, Sweden, and Heihe, China. **(A)** Rainfall in 2018 and historical average. **(B)** Average temperature in 2018 and historical average. **(C)** Day length. Data on rainfall and temperature in Uppsala and Heihe taken from https://www.accuweather.com. Data on Uppsala day length taken from https://www.timeanddate.com/sun/ and data on Heihe day length taken from https://richurimo.51240.com.

Table S1 | Rice lines/Varieties/Cultivars used for screening of adaptation

Lab id number	Line/Variety/Cultivar
1	Longdao 3
2	Longjing 2
3	Songjing 5
4	Dongnong 416
5	Hejiang 20
6	Jiandao 6
7	Kendao 12
8	Mudanjiang 27
9	Kendao 13
10	Songjing 1
11	Wuyoudao 1
12	Kendao 8
13	Shennong 265
14	Nipponbare
15	Songjing 3
16	Longjing 8
17	Song02-813
18	Heijing 5
19	Liaojing 5
20	Liaojing 326
21	Liaojing 454
22	Jijing 80
23	Changbai 15
24	Liaojing 294
